# Factors influencing the participation of gastroenterologists and hepatologists in clinical research

**DOI:** 10.1186/1472-6963-8-208

**Published:** 2008-10-08

**Authors:** Anouk T Dev, Teresa L Kauf, Amany Zekry, Keyur Patel, Karen Heller, Kevin A Schulman, John G McHutchison

**Affiliations:** 1 Duke Clinical Research Institute, PO Box 17969, Durham, North Carolina, USA; 2 Division of Gastroenterology, Department of Medicine, Duke University School of Medicine, Durham, North Carolina, USA; 3 Division of General Internal Medicine, Department of Medicine, Duke University School of Medicine, Durham, North Carolina, USA; 4 Monash University, Melbourne, Australia; 5 University of Florida, Gainesville, USA; 6 University of New South Wales, Sydney, Australia

## Abstract

**Background:**

Although clinical research is integral to the advancement of medical knowledge, physicians face a variety of obstacles to their participation as investigators in clinical trials. We examined factors that influence the participation of gastroenterologists and hepatologists in clinical research.

**Methods:**

We surveyed 1050 members of the American Association for the Study of Liver Diseases regarding their participation in clinical research. We compared the survey responses by specialty and level of clinical trial experience.

**Results:**

A majority of the respondents (71.6%) reported involvement in research activities. Factors most influential in clinical trial participation included funding and compensation (88.3%) and intellectual pursuit (87.8%). Barriers to participation were similar between gastroenterologists (n = 160) and hepatologists (n = 189) and between highly experienced (n = 62) and less experienced (n = 159) clinical researchers. These barriers included uncompensated research costs and lack of specialized support. Industry marketing was a greater influence among respondents with less trial experience, compared to those with extensive experience (15.7% vs 1.6%; *P *< .01). Hepatologists and respondents with extensive clinical trial experience tended to be more interested in phase 1 and 2 studies, whereas gastroenterologists and less experienced investigators were more interested in phase 4 studies.

**Conclusion:**

This study suggests that the greatest barrier to participation in clinical research is lack of adequate resources. Respondents also favored industry-sponsored research with less complex trial protocols and studies of relatively short duration.

## Background

Physicians who participate in clinical research contribute to the expanding knowledge base of medicine and have opportunities to offer their patients cutting-edge therapies. In addition, participation in clinical research is likely to benefit physicians by satisfying intellectual curiosity, increasing research opportunities, and aiding career advancement [1]. Previous studies have documented substantial barriers to participation, such as bureaucracy, lack of time, laboriousness, and insufficient financial reimbursement. These barriers prevent otherwise interested physicians from participating in research [2-4]. There is enormous potential in the biomedical sciences for translating new knowledge and technological capability into powerful tools for the prevention and treatment of disease. However, as recently stated by the Association of American Medical Colleges (AAMC) Clinical Research Summit, this potential is unlikely to be reached without greater participation in clinical research and a robust national program of research that "enjoys the participation and harnesses the full strength of all components of the health sector" [5].

Financial incentives have been shown to be among the most important factors motivating physician involvement in research [2,3,6,7]. In the United States, funding for biomedical research principally comes from industry and private sources. Currently, the National Institutes of Health and other government sources provide only 28% of research funding [8]. Clinical trials, and industry-sponsored trials in particular, which often carry greater reimbursement, are now viewed as essential sources of income for the maintenance of research programs and staff [9]. Recent research also indicates that academic-industry relationships in medicine have substantial benefits for industry sponsors and that the rate of industry support for clinical research is likely to increase [10]. Researchers in gastroenterology and hepatology will not be immune to this trend and may be forced to choose between conducting investigator-initiated research and that which is determined by industry interests [11,12]. There also could be a difference in clinical experience and participation rates between proceduralists (gastroenterologists) and nonproceduralists (hepatologists) and between physicians in private practice and those working predominantly in hospitals, as observed in previous studies [7,11]. In this study, we examined the factors that influence the participation of gastroenterologists and hepatologists in clinical research and identified the most important criteria in choosing to participate in competing trials.

## Methods

We developed a questionnaire to document respondents' demographic characteristics, factors that influence physician participation in research, factors important in enrolling patients in clinical trials, and respondents' preferences for types of research [see Additional file 1]. In addition, themes relating to barriers to participation and methods of financial reimbursement obtained from published literature were used to generate questions. An exploratory survey including these themes was given to a group of eight experienced physician-researchers in an open-ended or free-response format. The responses from this survey and ensuing discussion were used to generate fixed-alternative questions used in the final questionnaire. Face and content validity were increased by using different questions to elicit the same information.

A hypothetical case study was used to retest themes of study design and funding choices. The case study involved a patient with cirrhosis and hepatitis C who had not responded to standard pegylated interferon and ribavirin and was given three treatment options. Treatment option A involved a long-term clinical trial (5 years) of combination antiviral therapy (interferon and ribavirin) in varying doses designed to halt the progression of fibrosis. The trial was sponsored by a government institution with funding to cover the cost of the drug without reimbursement for additional costs. Treatment option B was an oral antiviral agent in phase 2 of investigation, given for a period of 6 months, and sponsored by a pharmaceutical company that funded all trial costs. Treatment option C was a 12-month trial of an intravenous antifibrotic agent in phase 3 of investigation, administered in varying doses including a nontreatment arm, and sponsored by a biotechnology company that funded all trial costs, including additional funds for support staff.

With the permission of the American Association for the Study of Liver Diseases (AASLD) executive, we obtained a register of physician members of the AASLD who had previously given consent to allow disclosure of their names to researchers. We mailed the questionnaire to all of these members (N = 1050). A second mailing to all registry members was conducted 12 weeks later to increase the response rate. Respondents could return the questionnaire by mail, fax, or e-mail, and they received no incentive for participation. This study was approved by the institutional review board of the Duke University Health System.

Descriptive characteristics of the respondents, preferences regarding clinical trial participation, funding, and patient enrollment, and responses to a patient case study and clinical trial scenarios were calculated as percentages of the total responses in each category. Differences between gastroenterologists and hepatologists, clinical researchers and non-clinical researchers, and physicians with limited and extensive clinical trial experience were also evaluated. Respondents who reported spending a nonzero amount of professional time on clinical research activities were considered to be clinical researchers. Extensive clinical trial experience was defined as participating in 5 or more clinical trials during the last 12 months. We used χ^2 ^tests or Fisher exact tests to make comparisons among groups, with *P *≤ .05 considered statistically significant.

Data from completed questionnaires were entered into a Microsoft Access database and analyzed using SAS software (SAS version 8.02, Cary, North Carolina, USA).

## Results

Characteristics of the 384 survey respondents (response rate, 37%) are shown in Table 1. Fifty percent of respondents practiced principally as hepatologists, and 42% practiced principally as gastroenterologists with an interest in hepatology. The majority of respondents (71.6%) reported involvement in research activities, over half (57.6%) reported involvement with clinical research, and 89.5% of these respondents reported clinical activities. Similarly, of those reporting involvement in clinical activities (academic or private practice), 70.1% also reported research activities.

**Table 1:** Characteristics of survey respondents (N = 384)*

Characteristic	All Respondents	Gastroenterologists	Hepatologists
Male	294 (79.3)	128 (83.7)†	138 (75.0)†
Race/ethnicity			
African American	13 (3.6)	4 (2.7)	9 (5.0)
Asian	68 (19.1)	29 (19.3)	34 (19.0)
Caucasian	241 (67.5)	101 (67.3)	124 (69.3)
Hispanic	19 (5.3)	11 (7.3)	4 (2.2)
Other	16 (4.5)	5 (3.3)	8 (4.5)
Age			
25 to 30 years	11 (2.9)	6 (3.8)	3 (1.6)
31 to 40 years	99 (26.2)	44 (28.0)	55 (29.4)
41 to 55 years	212 (56.1)	76 (48.4)	108 (57.8)
56 to 70 years	54 (14.3)	29 (18.5)	21 (11.2)
> 70 years	2 (0.5)	2 (1.3)	0 (0.0)
Specialty			
Gastroenterology	160 (41.7)		
Hepatology	189 (49.2)		
Other	29 (7.6)		
Degree			
MD or equivalent	343 (89.6)	149 (93.1)	169 (89.4)
MD and PhD	39 (10.2)	10 (6.3)	20 (10.6)
Other	1 (0.3)	1 (0.6)	0 (0.0)
Years in practice			
< 5 years	48 (13.3)	26 (17.7)†	17 (9.3)†
6 to 10 years	91 (25.3)	36 (24.5)	45 (24.7)
11 to 20 years	139 (38.6)	39 (26.5)†	89 (48.9)†
> 20 years	82 (22.8)	46 (31.3)†	31 (17.0)†
Practice			
Primary care	13 (3.7)	6 (4.1)	7 (3.9)
Tertiary care/academic facility	214 (60.8)	73 (50.0)†	119 (66.9)†
Community hospital	15 (4.3)	11 (7.5)†	3 (1.7)†
Private practice	98 (27.8)	52 (35.6)	45 (25.3)
Other	12 (3.4)	4 (2.7)	4 (2.3)
Professional activities‡			
Basic research	73 (19.0)	28 (17.5)	31 (16.4)
Clinical research	221 (56.7)	86 (53.8)†	116 (61.4)†
Academic research	115 (29.9)	31 (19.4)†	71 (37.6)†
Academic clinical practice	228 (59.4)	97 (60.6)	107 (56.6)
Private practice	166 (43.2)	86 (53.8)	72 (38.1)
Industry	19 (4.9)	5 (3.1)	13 (6.9)
Other	34 (8.9)	16 (10.0)	13 (6.9)

* Values are expressed as number (percentage) unless otherwise indicated. Some categories do not sum to 100% because of incomplete data. Of the 384 respondents, 349 identified their specialty as gastroenterology or hepatology, 1 as infectious disease, 2 as internal medicine, and 26 as other. Data were missing for 6 respondents.

† *P *≤ .05 for the comparison between specialties.

‡ Respondents could select more than 1 activity.

Respondents' experience with clinical trials is summarized in Table 2. Hepatologists were significantly more likely than gastroenterologists to report participation as an investigator in a clinical trial during the past 12 months (74.6% vs 59.4%; *P *< .01) and over the past 5 years (82.0% vs 72.5%; *P *< .05). Both gastroenterologists (67%) and hepatologists (76%) received the majority of their research funding from pharmaceutical companies; however, hepatologists had received more funding from universities/hospitals, government, and charitable organizations than gastroenterologists. Gastroenterologists selected pharmaceutical companies and government funding equally as their preferred source of funding, whereas hepatologists showed a clear preference for industry funding.

**Table 2:** Experience with clinical therapeutic trials among gastroenterologists and hepatologists*

Experience	Gastroenterologists (n = 160)	Hepatologists (n = 189)
Number of clinical trials in the previous 12 months		
0	65 (40.6)‡	48 (25.4)‡
1 to 4	70 (43.8)	89 (47.1)
5 to 10	17 (10.6)‡	43 (22.8)‡
> 10	7 (4.4)	8 (4.2)
Number of clinical trials in the previous 5 years		
0	44 (27.5)‡	34 (18.0)‡
1 to 4	40 (25.0)	34 (18.0)
5 to 10	35 (21.9)	42 (22.2)
> 10	40 (25.0)‡	79 (41.8)‡
Types of clinical trials		
Drug trials (human studies)	112 (42.9)	149 (57.1)
Basic science research	28 (17.5)‡	51 (27.0)‡
Non-drug trials (eg, procedural)	39 (24.4)	53 (28.0)
Non-drug epidemiological research	51 (31.9)‡	102 (54.0)‡
Other	1 (0.6)	3 (1.6)
Sources of funding for clinical trials		
Pharmaceutical company	107 (66.9)‡	144 (76.2)‡
Government institutions	54 (33.8)‡	96 (50.8)‡
Nongovernmental, charitable, or philanthropic organization	15 (9.4)‡	49 (25.9)‡
University or hospital funding	55 (34.4)‡	92 (48.7)‡
Other	1 (0.6)	2 (1.1)
Most preferred funding source†		
Pharmaceutical company	52 (44.8)	79 (50.1)
Government institution	46 (41.1)	59 (37.8)
Nongovernmental, charitable, or philanthropic organization	13 (12.2)	10 (7.0)
University or hospital funding	7 (6.4)	12 (8.1)
Other	0 (0.0)	1 (3.2)

* Values are expressed as number (percentage) unless otherwise indicated. Responses refer to clinical studies in which the respondent participated as an investigator.

† Indicates respondents who reported "most preferred" for each funding source.

‡ *P *≤ .05 for the comparison between specialties.

Table 3 shows factors influencing physician participation in clinical research by specialty and for clinical researchers and non-clinical researchers. Nearly 90% of all respondents cited level of funding or compensation commensurate with the time and effort associated with the clinical trial as extremely or very important in influencing the decision to participate. Other important considerations influencing participation in clinical research included intellectual pursuit and easy access to clinical trials. Hepatologists were especially likely to cite adequate funding or compensation levels (92.6% vs 85.0%; *P *< .05) and offers by sponsors to fund additional projects as influential factors (76.2% vs 65.6%; *P *< .05), compared with gastroenterologists. Clinical researchers were significantly more likely than non-clinical researchers to report sponsor-funded research (75.5% vs 66.1%; *P *= .05), peer recommendations (55.2% vs 38.0%; *P *< .001), and industry marketing (42.9% vs 11.8%; *P *< .0001) influenced their participation in clinical trials.

**Table 3:** Factors influencing participation in clinical trials, by specialty and current participation in clinical research*

Factor	Specialty		Clinical Research Participation	
	Gastroenterology (n = 160)	Hepatology (n = 189)	Clinical Researchers (n = 221)	Non-Clinical Researchers (n = 163)

Easy access to clinical trials or therapy	142 (88.8)†	152 (80.4)†	181 (81.9)	140 (85.9)
Level of funding or compensation for time and effort	136 (85.0)†	175 (92.6)†	196 (88.7)	143 (87.7)
Relationship with institution conducting the trial	121 (75.6)	142 (75.1)	163 (73.8)	123 (75.5)
Additional funding offers from sponsors	105 (65.6)†	144 (76.2)†	146 (66.1)†	123 (75.5)†
Recommendation from peers	90 (56.3)†	71 (37.6)†	84 (38.0)†	90 (55.2)†
Industry marketing	43 (26.9)	44 (23.3)	26 (11.8)†	70 (42.9)†
Intellectual pursuit	135 (84.4)	172 (91.0)	191 (86.4)	146 (89.6)

* Values are expressed as number (percentage) responding "extremely important" or "very important.

† *P *≤ .05 for the comparison between specialties or between clinical and non-clinical researchers.

Table 4 reports barriers to clinical research participation by specialty and for clinical researchers and non-clinical researchers. Over half of all respondents reported that excessive (uncompensated) research costs completely prevented participation in clinical research. About 40% reported that excess costs were of concern but did not prevent participation. In addition, 35.7% of physicians reported that lack of specialized support staff prevented them from participating in clinical research. Respondents who were not involved in clinical research were more than twice as likely as those who were involved to report lack of specialized support staff as a barrier to clinical trial participation (49.1% vs 23.5%; *P *< .001). Respondents were also concerned about the complexity of institutional review board (IRB) requirements and ethical considerations. Although few respondents reported that IRB requirements completely prevented them from participating in clinical trials, 68.7% of gastroenterologists and 74.3% of hepatologists listed IRB requirements as a concern.

**Table 4:** Factors preventing clinical trial participation, by specialty and current participation in clinical research*

Factor	Specialty		Clinical Research Participation	
	Gastroenterology (n = 160)	Hepatology (n = 189)	Clinical Researchers (n = 221)	Non-Clinical Researchers (n = 163)

Increasing complexity of trials	12 (8.1)	10 (5.4)	10 (4.7)	13 (8.4)
Excessive trial costs not covered by the trial sponsor	76 (50.7)	98 (52.7)	115 (54.5)†	73 (46.5)
Complexity of Institutional Review Board requirements	20 (13.3)	10 (5.4)	18 (8.5)	15 (9.6)
Inferior trial medication(s) compared to standard therapy	65 (43.3)	86 (46.0)	115 (54.0)†	54 (34.6)
Lack of specialized support staff	50 (32.9)	71 (38.0)	52 (24.4)†	80 (50.6)
Concern about sponsor control of trial decision-making, data, publication, etc	22 (14.8)	37 (19.9)	54 (25.6)†	18 (11.5)
Too busy with clinical practice commitments	37 (24.3)	46 (24.7)	14 (6.6)†	74 (46.8)
Ethical considerations	41 (27.2)	45 (24.1)	70 (32.9)†	26 (16.6)
Difficulty in accessing the appropriate patient population	15 (9.9)	18 (9.6)	25 (11.7)†	13 (8.3)
Not interested in participating in sponsored clinical research	14 (9.4)†	22 (11.9)	7 (3.3)†	32 (20.5)

* Values are expressed as number (percentage) responding "completely prevents me [from participating]."

† *P *≤ .05 for the comparison between specialties or between clinical and non-clinical researchers.

One-fourth of all respondents reported that ethical considerations completely prevented them from participating in clinical trials, and nearly half (49.1%) said they were concerned about trial ethics. As might be expected, a substantial majority of responding physicians (80.7%) were concerned about the inferiority of study medications compared to standards of care. Many respondents (62.4%) were concerned about sponsor control, and 17.6% of all responding physicians reported that concerns about sponsor control of decision making, data, and/or publication completely prevented them from participating in clinical research. Interestingly, clinical researchers were more likely to report concerns about study medications (52.0% vs 33.1%; *P *< .001) and sponsor control (24.4% vs 11.0%; *P *< .001) as barriers to clinical trial participation, compared to respondents who were not involved in clinical research. Barriers to clinical trial participation were similar for both highly experienced and less experienced clinical researchers, although the former were more likely to cite concerns about study medications as a barrier (64.5% vs 47.2%; *P *= .02). Finally, a quarter of all respondents and 45.4% of those not involved in clinical research reported that clinical practice commitments did not leave enough time to participate in clinical studies.

**Figure 1:**
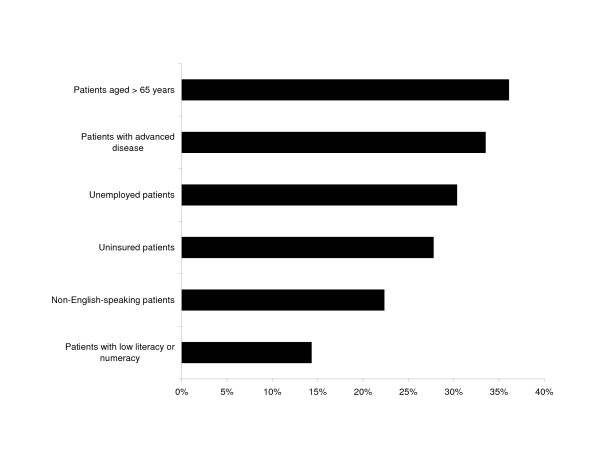
Proportion of respondents very likely to enroll patients in cinical research by patient characteristics.

The perceived ability of the patient to comply with, adhere to, and comprehend protocols was cited by at least 90% of all respondents as extremely or very important in influencing the decision to enroll patients in clinical trials. Cost considerations, either to the patient or the physician, were also frequently cited as factors influencing enrollment. A minority of physicians were likely to enroll patients with high-risk characteristics such as older age, advanced disease, or lack of insurance in clinical trials (Figure 1). Gastroenterologists were more likely than hepatologists to cite logistical considerations (55% vs 43.9%; *P *< .05) and cost to the patient (85.6% vs 76.2%; *P *< .05) as affecting patient enrollment decisions.

Respondents' interest in trials with specific characteristics is summarized in Table 5. Overall, respondents were most interested in shorter trials (duration less than 2 years) and phase 3 trials. Less than half of the respondents reported a preference for phase 2 or phase 4 trials, and fewer than one-third were very interested in phase 1 studies. Interest in longer trials and in placebo-controlled trials or trials with nontreatment arms was low. Only 1 in 5 respondents reported interest in trials involving multiple treatment arms and/or crossover designs or trials involving invasive procedures.

**Table 5:** Characteristics of preferred clinical trials*

Characteristic	Specialty		Experience	
	Gastroenterology (n = 160)	Hepatology (n = 189)	Less Experienced Researchers (n = 159)	More Experienced Researchers (n = 62)

Phase 1 trials	36 (22.5)†	73 (38.6)†	59 (37.1)†	32 (51.6)†
Phase 2 trials	64 (40.0)†	107 (56.6)†	91 (57.2)†	52 (83.9)†
Phase 3 trials	97 (60.6)	108 (57.1)	114 (71.7)†	53 (85.5)†
Phase 4 trials	76 (47.5)†	68 (36.0)†	92 (57.9)	28 (45.2)
Duration ≤ 2 years	100 (62.5)	126 (66.7)	127 (79.9)†	58 (93.6)†
Duration > 2 years	38 (23.8)	59 (31.2)	57 (35.9)†	35 (56.5)†
Trials with placebo or no treatment arm	51 (31.9)	55 (29.1)	68 (42.8)	28 (45.2)
Trials involving invasive procedures	40 (25.0)†	30 (15.9)†	44 (27.7)	18 (29.0)
Multiple-arm and crossover protocols	36 (22.5)	35 (18.5)	40 (25.2)†	24 (38.7)
Industry funding	76 (47.5)	104 (55.0)	86 (54.1)†	50 (80.7)†
Government funding	78 (48.8)†	121 (64.0)†	104 (65.4)†	56 (90.3)†
Non-government funding	78 (48.8)	103 (54.5)	96 (60.4)†	51 (82.3)†

* Values are expressed as number (percentage) responding "very interested".

† *P *≤ .05 for the comparison between specialties or between clinical and non-clinical researchers.

Physician interest in participating in various types of clinical studies was strongly associated with specialty and clinical trial experience. Hepatologists were more interested than gastroenterologists in participating in phase 1 studies (38.6% vs 22.5%; *P *< .001) and phase 2 studies (56.6% vs 40%; *P *< .01). However, gastroenterologists were more interested than hepatologists in phase 4 studies (47.5% vs 36.0%; *P *< .05). Phase 3 studies appeared to be the most desired phase of study among both gastroenterologists and hepatologists. These preferences were similar among experienced clinical researchers compared to less experienced researchers.

**Figure 2:**
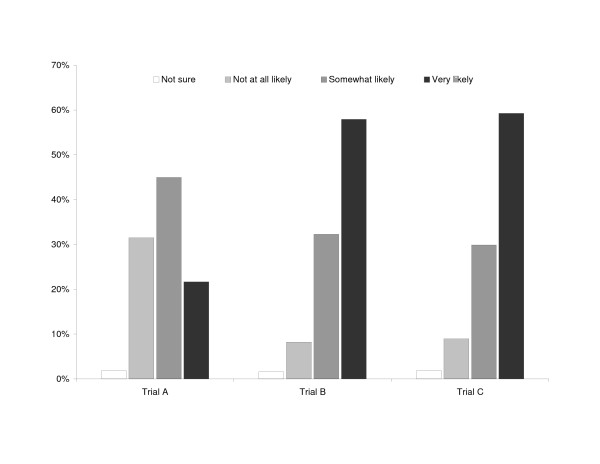
Responses to the patient case study.

Results from the hypothetical patient enrollment scenarios (Figure 2) are consistent with preferences for shorter trials and concerns about funding noted above. Significantly fewer respondents reported that they would be very or somewhat likely to enroll the patient into trial A, compared to trials B and C (65.6% vs 88.8% vs 87.8%; respectively; *P *< .001). This pattern was consistent across specialty, current participation in clinical research, and clinical trial experience.

## Discussion

Previous work examining physician participation in clinical research has focused for the most part on oncology, internal medicine, and general practice [3,7,13-17]. In this study, we explored hepatologists' and gastroenterologists' perceptions of barriers to clinical investigation in the current practice environment. The majority of respondents were involved in research activities and indicated intellectual pursuit as an important factor in influencing participation in research. However, regardless of the level of clinical trial experience, respondents cited issues with resources as barriers to participation in clinical research, including financial compensation, IRB support, availability of specialized support staff, and the complexity of clinical trials. This lack of support for core research infrastructure was reflected in concerns about ability to cover the total costs of clinical research in an increasingly resource-constrained practice environment.

Other physician specialists, particularly oncologists, have identified the same barriers to clinical trial participation cited by gastroenterologists and hepatologists in the present study [3,13-15]. Although one study of breast cancer specialists indicated that physicians have become more amenable to enrolling patients into clinical trials [14], organizational support and resource costs remained significant concerns. Our findings suggest that these concerns are not limited to oncology, thus inviting speculation that the same factors might apply to other physician specialists. However, given the paucity of related research in other specialties and the limitations of existing studies, definitive conclusions about barriers to clinical trial participation across specialties are difficult to make.

Clinical research is the cornerstone of evidence-based medical practice and the key translational step from basic science discovery to benefits for individual patients. This survey, the largest of its kind in this field, illustrates the interest in clinical research among members of the AASLD. However, it also describes some worrisome trends where physicians preferred participation in typical short-term clinical trials over more definitive trial designs and participation was influenced by the level of payment. Hepatologists in particular showed a clear preference for industry funding. Hepatologists were also more likely to have participated in a clinical trial over the preceding 5 years, and their responses may reflect the realities of conducting research in the current climate, in which government funding is in decline and the reimbursement linked to industry-sponsored trials has become crucial to the maintenance of research programs [8,9]. A review of industry funding in gastrointestinal clinical research confirms that industry sponsorship of research is increasing and, therefore, will continue to determine the research agenda [12].

Hepatologists' greater participation in clinical trials and their preference for industry-sponsored studies also may reflect a broader dynamic related to the structure of physician compensation. In the United States, reimbursement for many medical procedures has been relatively higher – on an hourly basis – than for evaluation and management services [18,19], though that gap has been reduced by changes in the Medicare fee schedule over time [20]. If reimbursement levels in hepatology – where the focus is less procedural than for gastroenterology as a whole – are inadequate to support physician practices, hepatologists may feel pressured to participate in industry-sponsored clinical trials as a means of supplementing their practice income and/or supporting academic salaries.

Anecdotal evidence suggests that another possible explanation for the greater participation of hepatologists in clinical trials is that gastroenterologists, in general, have been more exposed to device-oriented research and its marketing, which has remained relatively constant during the past decade. In contrast, hepatologists have been exposed to many new companies, sponsors, and trials for hepatitis and other liver diseases during this time, particularly during the past 5 to 10 years. Thus, hepatologists' exposure to, and requests to participate in, clinical studies may have been greater, and perhaps their incentives higher, than those for gastroenterologists.

Although industry sponsorship was the respondents' preferred source of funding, relationships with sponsors, especially regarding sponsor control over trial decision making, data, and/or publication, were a major concern, reflecting ongoing explorations of the means of assuring transparency in reporting the results of clinical research. Less experienced investigators, in particular, were more likely to be influenced by industry marketing when considering competing research opportunities. Encouragingly, a recent review of gastrointestinal research publications reported that, on average, industry-sponsored studies were of superior methodologic quality to studies funded by other sources and were no more likely than other studies to publish results that favored the study sponsor, although an extremely high percentage of all studies in these journals reported positive results [12].

This study has some limitations. Our findings may not be representative of all gastroenterologists and hepatologists eligible to conduct clinical research in the United States. We targeted members of the AASLD under the assumption that most gastroenterologists and hepatologists with an interest in clinical research likely would be associated with the organization. It is reasonable to expect that physicians who perceive high barriers to participation in such activities would be less likely to join professional, research-focused organizations. Thus, our focus on AASLD members may have introduced a bias toward those facing fewer barriers to clinical research participation. In that sense, the results of our study may be interpreted as a conservative estimate of barriers to the conduct of clinical research in the United States.

Previous participation as an investigator in clinical studies was not a prerequisite for survey participation. Limiting our sample only to physicians with current or previous participation in clinical research, as other surveys have done [15], also may have introduced a bias into the study. Further, our definition of clinical research experience was based on respondents' self-reports of participation and did not consider whether respondents accrued patients to those studies in which they agreed to participate. It is possible that physicians who agree to participate in clinical trials but do not accrue patients may perceive (and report) barriers to clinical research differently than those with greater enrollment success. These participants were more likely to be involved in research activity as opposed to non members, thereby creating an additional bias.

Within the AASLD membership, only members who had previously given consent to allow disclosure of their names to researchers were contacted for participation in this study; this also may have introduced bias. Moreover, not all AASLD members in the target population completed the survey. The overall response rate was comparative to other mail surveys of physicians and other health care professionals and perhaps could have been improved through the use of an incentive [21-27]. Finally, our survey may not have captured all aspects of clinical trial participation relevant to gastroenterologists and hepatologists.

## Conclusion

This study suggests that the greatest barrier to participation in clinical research is a lack of adequate resources, which most likely influenced the preference for less complex trial protocols and studies of relatively short duration. The findings of the study also reflect the reality of industry sponsorship of much clinical investigation in gastroenterology and hepatology and highlight some of the critical gaps in the clinical research infrastructure [28] that may ultimately limit the expansion of evidence-based clinical practice and translational research. One way to close gaps in the research infrastructure is for large institutions (and perhaps smaller ones) to create centralized research support services. Though challenging to implement at a reasonable cost, such services may provide a more effective research environment that will improve patient safety, increase economic and medical efficiency, and provide a more standardized and regulatory-compliant process for conducting research.

## Abbreviations

AAMC: Association of American Medical Colleges; AASLD: American Association for the Study of Liver Diseases; IRB: institutional review board.

## Competing interests

Drs Schulman and McHutchison have received grants and/or consultancy fees from a number of private organizations. They have made available online detailed listings of financial disclosures . All other authors declare that they have no competing interests.

## Authors' contributions

ATD and TLK conceived and designed the study, acquired the data, analyzed and interpreted the data, and drafted the manuscript. AZ designed the study, acquired the data, analyzed and interpreted the data, and revised the manuscript for important intellectual content. KP designed the study, acquired the data, analyzed and interpreted the data, and revised the manuscript for important intellectual content. KH acquired the data, analyzed and interpreted the data, and revised the manuscript for important intellectual content. KAS conceived and designed the study, analyzed and interpreted the data, and revised the manuscript for important intellectual content. JGM conceived and designed the study, analyzed and interpreted the data, revised the manuscript for important intellectual content, and supervised the study. All authors read and approved the final manuscript.

## Additional contributions

We thank Damon Seils of Duke University for assistance with manuscript preparation. Mr Seils did not receive compensation for his assistance apart from his employment at the institution where the study was conducted.

## Pre-publication history

The pre-publication history for this paper can be accessed here:



## Supplementary Material

Additional file 1 **Survey of involvement and participation in clinical trials.** The questionnaire administered to study participants. Click here for file
