# Supplementary material for: Factors influencing the participation of gastroenterologists and hepatologists in clinical research
Source: BMC Health Serv Res. 2008 Oct 8;8:208. doi: 10.1186/1472-6963-8-208 (PMC2572062; doi:10.1186/1472-6963-8-208)
Supplement: Additional file 1 — Survey of involvement and participation in clinical trials. The questionnaire administered to study participants. [file 1472-6963-8-208-S1.pdf]

Appendix. Survey of Involvement and Participation in Clinical Trials

Physicians are frequently asked to participate in clinical research and are increasingly presented with a wide variety of research projects from which to choose. This questionnaire aims to define the factors which influence hepatologists to participate in clinical research.

I. DEMOGRAPHIC AND PRACTICE INFORMATION

**1. Gender** Male ☐ Female ☐	**2. Race/ Ethnicity (Optional)**<br>African American ☐<br>Asian ☐<br>Caucasian ☐<br>Hispanic ☐<br>Other (please specify) ______ ☐
**3. Age**<br>25-30 years ☐<br>31-40 years ☐<br>41- 55 years ☐<br>56-70 years ☐<br>>70 years ☐	
**4. Specialty**<br>Gastroenterologist ☐<br>Hepatologist ☐<br>Infectious Diseases specialist ☐<br>Internal Medicine specialist ☐<br>Other (please specify) ______ ☐	**5. Practice setting**<br>Primary Care Center ☐<br>Tertiary Care/ Academic Facility ☐<br>Community Hospital ☐<br>Private Practice ☐<br>Other (please specify) ______ ☐
**6. Current degree**<br>MD or equivalent ☐<br>PhD ☐<br>MD/PhD ☐<br>Other (please specify) ______ ☐	**7. Years in practice**<br>< 5 years ☐<br>6 – 10 years ☐<br>11 – 20 years ☐<br>> 20 years ☐

8. Please indicate the percentage of time you spend on each of the following professional activities:

	0%	1 – 25%	26 – 50%	51 – 75%	> 75%
Research-Basic	☐	☐	☐	☐	☐
Research-Clinical	☐	☐	☐	☐	☐
Research-Academic	☐	☐	☐	☐	☐
Clinical- academic	☐	☐	☐	☐	☐
Clinical-Private-Practice	☐	☐	☐	☐	☐
Industry	☐	☐	☐	☐	☐
Other (please specify) ______	☐	☐	☐	☐	☐

II. EXPERIENCE WITH CLINICAL THERAPEUTIC TRIALS

**9. How many clinical trials have you participated in as an investigator during the last <u>12 months</u>?**	none ☐	1 – 4 trials ☐	5 – 10 trials ☐	>10 trials ☐
**10. How many clinical trials have you participated in as an investigator during the last <u>5 years</u>?**	none ☐ (skip to Q16)	1 – 4 trials ☐	5 – 10 trials ☐	>10 trials ☐

11. Please indicate the type(s) of clinical trials you have participated in as an investigator:

- Drug trials (Human studies) ☐
- Basic Science Research ☐
- Non-drug trials (eg. Procedural) ☐
- Non-drug epidemiological research ☐
- Other (please specify) ______ ☐

12. Please indicate the funding source for clinical trials you have participated in as an investigator:

- Pharmaceutical company ☐
- Government institutions ☐
- Non government/ charitable/ philanthropic agencies ☐
- University/ Hospital funding ☐
- Other (please specify) ______ ☐

13. Please indicate in order from 1 to 5 your preference for source of funding for trials (1 = most preferred, 5 = least preferred or N/A):

- Pharmaceutical company ___
- Government institutions ___
- Non government/ charitable/ philanthropic agencies ___
- University/ Hospital funding ___
- Other (please specify) ______ ___

14. Regardless of whether you currently participate in clinical trials or not, please indicate the importance of the factors below in influencing your decision to enroll patients in clinical trials.

	Extremely important	Very Important	Somewhat important	Not at all important	Not sure
Patient's level of education	☐	☐	☐	☐	☐
Patient's race/ethnicity	☐	☐	☐	☐	☐
Patient's socioeconomic status	☐	☐	☐	☐	☐
Patient's ability to comprehend trial protocols	☐	☐	☐	☐	☐
Pressure from patient / patient's family members	☐	☐	☐	☐	☐
Concern about losing patient to follow up	☐	☐	☐	☐	☐
Anticipated logistical problems (eg travel)	☐	☐	☐	☐	☐
Severity of disease	☐	☐	☐	☐	☐
Cost incurred by you or your department	☐	☐	☐	☐	☐
Cost incurred by the patient	☐	☐	☐	☐	☐
Patient's inability to comply or adhere to trial protocol	☐	☐	☐	☐	☐

15. How important are the factors below in influencing your participation in clinical trials?

	Extremely important	Very Important	Somewhat important	Not at all important	Not sure
Easy access to clinical trials/therapy	☐	☐	☐	☐	☐
Level of funding/ equitable compensation for time and effort associated with the trial	☐	☐	☐	☐	☐
Relationship or previous experience with the institution conducting the trial	☐	☐	☐	☐	☐
Offer from sponsors to fund additional projects/ establish a research fund etc.	☐	☐	☐	☐	☐
Recommendation from peers	☐	☐	☐	☐	☐
Industry marketing	☐	☐	☐	☐	☐
Intellectual pursuit	☐	☐	☐	☐	☐

16. Which factors prevent you from participating in clinical trials:

	Completely prevents me	Is a concern, but doesn't prevent me from participating	Not a concern	Not sure
Increasing complexity of trials	☐	☐	☐	☐
Excessive trial costs not covered by the trial sponsor	☐	☐	☐	☐
Complexity of Institutional Review Board requirements	☐	☐	☐	☐
Inferior trial medication(s) compared to standard therapy	☐	☐	☐	☐
Lack of specialized support staff	☐	☐	☐	☐
Concern about sponsor control of trial decision-making, data, publication, etc	☐	☐	☐	☐
Too busy with clinical practice commitments	☐	☐	☐	☐
Ethical considerations	☐	☐	☐	☐
Difficulty in accessing the appropriate patient population	☐	☐	☐	☐
Not interested in participating in sponsored clinical research	☐	☐	☐	☐

17. What is the likelihood of you enrolling the following patients in clinical therapeutic trials?

	Very likely	Somewhat likely	Not at all likely	Not sure
Non English speaking patients	☐	☐	☐	☐
Patients > 65 yrs	☐	☐	☐	☐
Unemployed patients	☐	☐	☐	☐
Patients with reduced numeracy and literacy skills	☐	☐	☐	☐
Patients with advanced disease	☐	☐	☐	☐
Uninsured patients	☐	☐	☐	☐

18. Please indicate your level of interest for participating as an investigator in the following trials:

	Very interested	Somewhat interested	Not at all interested	Not sure
Phase I trials (Initial introduction of an investigational new drug or pilot study to establish a safe dose range)	☐	☐	☐	☐
Phase II trials (Controlled clinical studies conducted to evaluate the effectiveness of a drug for a particular condition)	☐	☐	☐	☐
Phase III trials (pre-marketing controlled studies to determine safety, efficacy and appropriate dosage for a particular condition)	☐	☐	☐	☐
Phase IV trials (Post marketing or open label studies to delineate additional information about drug risks, benefits and optimal use)	☐	☐	☐	☐
Trials with a study duration = 2 yrs	☐	☐	☐	☐
Trials with a study duration > 2 yrs	☐	☐	☐	☐
Trials with a placebo/ non treatment arm	☐	☐	☐	☐
Trials involving invasive procedures	☐	☐	☐	☐

Trials with multiple arms and crossover protocols	☐	☐	☐	☐
Trials funded by pharmaceutical companies	☐	☐	☐	☐
Trials funded by government agencies	☐	☐	☐	☐
Trials funded by non government agencies	☐	☐	☐	☐

19. Consider a 45 year old male with Hepatitis C cirrhosis (genotype 1) who is unresponsive to pegylated interferon and ribavirin therapy and requests further treatment. How likely are you to enroll this patient in each of these trials:

A. A multi-center trial involving long term combination therapy for 5 years with the aim of reversing fibrosis. The trial includes multiple arms with varying doses. The trial is sponsored by a government institution which only funds the cost of the trial drug without reimbursement for additional costs.

Very likely	Somewhat likely	Not at all likely	Not sure
☐	☐	☐	☐

B. A 6-month trial involving an oral agent with antiviral effects recommended for non-responders to standard therapy in phase II of clinical trial investigation. The trial includes multiple arms with varying doses. No significant side-effects were reported in phase I of drug evaluation. The trial is sponsored by a pharmaceutical company which funds all trial costs.

Very likely	Somewhat likely	Not at all likely	Not sure
☐	☐	☐	☐

C. A 12-month trial of an intravenous agent with anti-fibrotic effects in phase III of development. Minimal side-effects were reported in phase I and II of drug evaluation. The trial includes multiple arms with varying doses and a non-treatment arm. The trial is sponsored by a biotechnology company which funds all trial costs and also provides additional funds for support staff.

Very likely	Somewhat likely	Not at all likely	Not sure
☐	☐	☐	☐

Thank you for your participation.
